# The Association of Ankle Brachial Index, Protein-Energy Wasting, and Inflammation Status with Cardiovascular Mortality in Patients on Chronic Hemodialysis

**DOI:** 10.3390/nu9040416

**Published:** 2017-04-21

**Authors:** Hideki Ishii, Hiroshi Takahashi, Yasuhiko Ito, Toru Aoyama, Daisuke Kamoi, Takashi Sakakibara, Norio Umemoto, Yoshitaka Kumada, Susumu Suzuki, Toyoaki Murohara

**Affiliations:** 1 Department of Cardiology, Nagoya University Graduate School of Medicine, 65, Tsurumai-cho, Showa-ku, Nagoya 466-8550, Japan; susumusuzuki@med.nagoya-u.ac.jp (S.S.); murohara@med.nagoya-u.ac.jp (T.M.); 2 Department of Nephrology, Fujita Health University School of Medicine, Toyoake 470-1192, Japan; hirotaka@fujita-hu.ac.jp; 3 Department of Nephrology and Renal Replacement Therapy, Nagya University Graduate School of Medicine, Nagoya 466-8550, Japan; yasuito@med.nagoya-u.ac.jp; 4 Cardiovascular Center, Nagoya Kyoritsu Hospital, Nagoya 466-8550, Japan; taoyama@kaikou.or.jp (T.A.); dkamoi@kaikou.or.jp (D.K.); Salcst7@gmail.com (T.S.); kisyuume2000@yahoo.co.jp (N.U.); 5 Department of Cardiovascular Surgery, Matsunami General Hospital, Kasamatsu 501-6062, Japan; Ykumada1206@gmail.com

**Keywords:** hemodialysis, ankle brachial index, protein energy wasting, geriatric nutritional risk index, inflammation

## Abstract

Protein-energy wasting (PEW) is highly prevalent in hemodialysis (HD) patients. We investigated the association of abnormal ankle brachial index (ABI), PEW, and chronic inflammation status with clinical prognosis in HD patients. A total of 973 HD patients were enrolled and were followed-up for 8 years. As a marker of the PEW, geriatric nutritional risk index (GNRI) was used. Cut-off levels were 91.2 for GNRI defined from previous studies and 1.9 mg/L for C-reactive protein (CRP) as median value, respectively. Abnormal ABI was seen in 332 (34.1%) patients. Declined GNRI and elevated CRP levels were independently associated with abnormal ABI (odds ratio (OR) 0.97, 95% confidence interval (CI) 0.96–0.99, *p* = 0.0009 and OR 1.40, 95% CI 1.07–1.83, *p* = 0.013, respectively). GNRI levels were also independently correlated with CRP levels (β = −0.126, *p* < 0.0001). During follow-up period, 283 (29.1%) patients died, including 123 (12.6%) due to cardiovascular disease (CVD). Abnormal ABI (adjusted hazard ratio (HR) 1.62, 95% CI 1.13–2.32, *p* = 0.0096), GNRI < 91.2 (adjusted HR 1.57, 95% CI 1.06–2.33, *p* = 0.023) and CRP > 1.9 mg/L (adjusted HR 1.89, 95% CI 1.31–2.77, *p* = 0.0007) independently predicted mortality due to CVD, respectively. In conclusion, abnormal ABI, GNRI, and CRP levels were closely associated with each other, and the combination of these variables increase their predictive values for the risk of mortality due to CVD and all-cause mortality in HD patients.

## 1. Introduction

Patients on hemodialysis (HD) are at an extremely high risk of cardiovascular disease [1,2]. The incidence of cardiovascular disease in patients on HD is reportedly 20–30 higher times compared to non-HD population [3]. Therefore, a predictive value to detect clinical outcomes, particularly cardiovascular diseases in HD patients has been warranted. 

In the clinical settings, peripheral artery disease (PAD) is frequently seen in patients on hemodialysis (HD) [4]. Additionally, the presence of PAD greatly affects clinical outcomes in such population [5]. In the clinical settings, ankle brachial index (ABI) has been widely used to detect PAD [6]. 

On the other hand, malnutrition and inflammation are highly prevalent in HD population. These association are characteristics for protein energy wasting (PEW) [7]. Recently, geriatric nutritional risk index (GNRI) has been developed as a simplified screening tool to assess not only the nutritional but also inflammatory status in HD patients [8]. 

In the study, we investigated the association of abnormal ABI, GNRI, and chronic inflammation status detected by C-reactive protein (CRP) levels with clinical prognosis in HD patients. In addition, we explored whether combination of these factors can more accurately predict clinical outcomes in such a high-risk population.

## 2. Materials and Methods

### 2.1. Enrolled Subjects

We enrolled a total of 973 clinically stable outpatients stably undergoing maintenance HD therapy for at least a month in Nagoya Kyoritsu Hospital with three associated clinics in Japan: the Central Clinic, the Ama Kyoritsu Clinic, and the Meiko Kyoritsu Clinic, respectively. To them, ABI, GNRI, and CRP levels were measured from January in 2007 to April in 2008. In this retrospective, the observational follow-up study, we investigated the association of ABI, PEW, and chronic inflammation status with mortality due to cardiovascular disease (CVD) in HD patients, and enrolled subjects were followed-up for 8 years. In advance, those with malignancy and/or active inflammatory diseases were excluded from the study. In the study, hypertension was defined as pre-dialysis systolic blood pressure >160 mm Hg and/or diastolic blood pressure >90 mm Hg, or a history of anti-hypertensive treatment [9]. Diabetes was defined as a history or presence of diabetes and/or if patients had a fasting plasma glucose concentration of ≥126 mg/dL or a glycosylated hemoglobin (HbA1c) concentration of >6.5%. Dyslipidemia was defined if patients had total cholesterol levels >220 mg/dL and/or lipid-lowering therapy. Smoking was defined either as a current smoker or as having discontinued cigarette use by 6 months before enrollment.

The physicians obtained written informed consent from each patient, and the study was approved by the hospital ethics committee. The results have not been published previously except in abstract form.

### 2.2. Endpoints

The endpoints were mortality due to CVD and all-cause mortality. Mortality due to CVD was defined as death due to cerebrovascular events, myocardial infarction, heart failure, and/or arrhythmia, other cardiovascular related death, or sudden death for unknown reasons. Clinical data were obtained from hospital charts and telephone interviews with patients by trained reviewers blinded to patients’ clinical backgrounds. ABI, which simultaneously measures systolic blood pressure in the arm (without dialysis access) and ankle blood pressure, was measured by using a Colin Waveform analyzer (form PWV/ABI; Colin medical Technology, Komaki, Japan). Abnormal ABI was defined by the following criteria: ABI < 0.9 or ABI > 1.4. As a marker of the PEW, geriatric nutritional risk index (GNRI) was calculated as follows: GNRI = (14.89 × albumin) + (41.7 × (body weight/body weight at BMI of 22)) [10]. Serum C-reactive protein (CRP) levels were also measured using a latex-enhanced high-sensitive CRP immunoassay. Cut-off levels were 91.2 for GNRI defined from a previous study [10] and 1.9 mg/L for CRP as median value, respectively. For laboratory data, fasting blood samples from A-V fistula were obtained before the first dialysis session in a week.

### 2.3. Statistics

In the study, we used SAS 6.10 software (SAS Institute, Cary, NC, USA) for statistical analyses. Variables with a normal distribution are expressed as mean values ± SD, and asymmetrically distributed data are given as median and interquartile range. Differences among the groups were evaluated by one-way analysis of variance (ANOVA) or Kruskal–Wallis test for continuous variables and by the chi-square test for categorical variables. Associated factors for abnormal baseline ABI were identified by use of the logistic model, and the multivariate model included all baseline valuables with *p* < 0.05 by univariate analysis. Relationship between GNRI and baseline variables were evaluated by multivariate regression analysis.

Differences in event-free survival among the groups with risk factors, such as an abnormal ABI, a GNRI < 91.2, and a CRP > 1.9 mg/L, were examined with the Kaplan–Meier method and compared using a log-rank test. Hazard ratios (HR) and 95% confidence intervals (CI) were calculated for each factor by a Cox proportional hazards analysis. In addition, to determine independent predictors for the endpoints, all baseline variables with *p* < 0.05 by univariate analysis were entered into a multivariate model. We calculated the C-index for receiver-operating characteristics curve, the net reclassification improvement (NRI) and the integrated discrimination improvement (IDI) to assess whether the values for predicting endpoints improved after the addition of an abnormal ABI, a GNRI < 91.2, and a CRP > 1.9 mg/L to a baseline model with established risk factors, which were baseline variables with *p* < 0.05 by univariate analysis, such as male sex, age, diabetes, previous CAD, previous stroke, hemoglobin, and creatinine.

Differences were considered significant at *p* < 0.05.

## 3. Results

### 3.1. Baseline Characteristics

Baseline characteristics in study population are shown in Table 1. Abnormal ABI (<0.9 or >1.4) was seen in 332 (34.1%) patients. Baseline GNRI and CRP levels were 94.1 ± 8.8 and 1.6 (0.8–5.0) g/dL, respectively. Declined GNRI and elevated CRP levels were independently associated with an abnormal ABI (odds ratio (OR) 0.97, 95% CI 0.96–0.99, *p* = 0.0009 and OR 1.40, 95% CI 1.07–1.83, *p* = 0.013, respectively) (Table 2). CRP levels were also independently correlated with GNRI levels (β = −0.138, *p* < 0.0001) (Table 3).

### 3.2. Follow-Up

**Figure 1:**
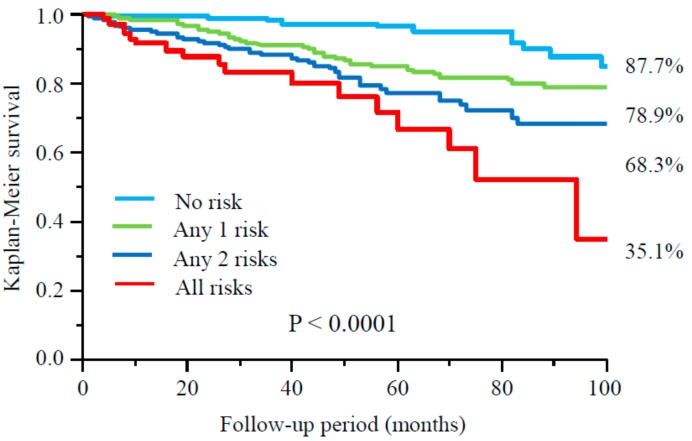
Cardiovascular survival among groups according to number of risk factors, abnormal ABI, GNRI, and CRP.

**Figure 2:**
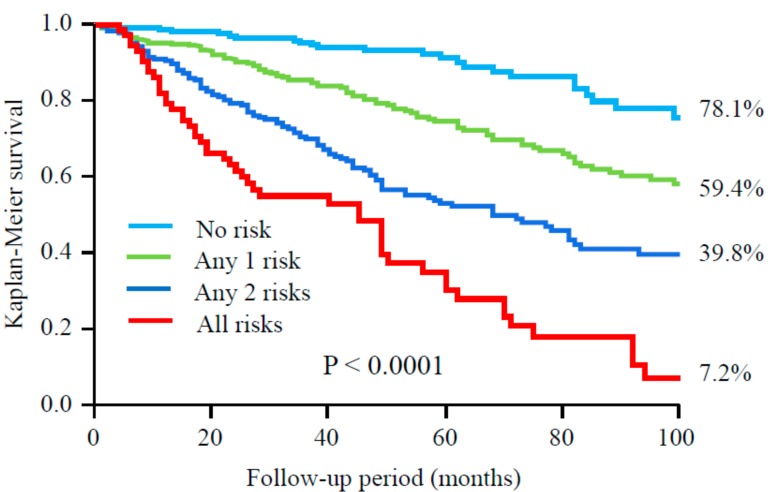
All-cause survival among groups according to number of risk factors, abnormal ABI, GNRI, and CRP.

During follow-up period (median 46 months), 39 patients moved to other institutions and 9 underwent kidney transplantation, and they were censored at the point of dropping out. A total of 283 (29.1%) patients died including 123 (12.6%) due to CVD. An abnormal ABI (adjusted HR 1.62, 95% CI 1.13–2.32, *p* = 0.0096), a GNRI <91.2 (adjusted HR 1.57, 95% CI 1.06–2.33, *p* = 0.023), and a CRP >1.9 mg/L (adjusted HR 1.89, 95% CI 1.31–2.77, *p* = 0.0007) were independent predictors for mortality due to CVD, respectively (Table 4). Furthermore, when patients were divided into groups according to number of these three risk factors, 8-year freedom from mortality due to CVD was 87.7%, 78.9%, 68.3%, and 35.1% among groups with no risk factor, any single risk factor, any two risk factors, and all risk factors, respectively (*p* < 0.0001) (Figure 1). As for all-cause mortality, a similar tendency was also seen among four groups (*p* < 0.0001) (Figure 2). Patients with all risk factors had 5.26-fold (95% CI 2.51–11.3, *p* < 0.0001) higher risk for mortality due to CVD compared to those without any risk factor after adjustment for other confounders. Similar results were obtained in all-cause mortality (adjusted HR 7.97, 95% CI 4.88–13.2, *p* < 0.0001) (Table 5).

To determine model discrimination, the C-index was calculated. Adding both ABI and GNRI significantly increased the C-index to predict mortality due to CVD, compared to a combination of established risk factors only. The C-index for all-cause mortality was higher in a baseline model with either ABI, GNRI, or CRP levels than in the baseline model alone (Table 6). 

## 4. Discussion

In the study, we found that the combination of ABI, GNRI, and CRP levels strongly increase their predictive values for mortality due to CVD and all-cause mortality risk in HD patients. Because HD patients have high prevalence of cardiovascular events, simple methods to predict clinical outcome in the HD population. From this perspective, our findings are of great significance. In addition, declined GNRI and elevated CRP levels were associated with abnormal ABI in HD patients.

Malnutrition status is now considered as one of independent predictors of poor prognosis in high-risk subjects such as the elderly population [11] and those with chronic heart failure [12]. As for patients with chronic kidney disease, PEW, which represents a state of decreased body stores of protein and energy fuels, is frequently seen in the clinical settings, and there is a strong relationship between PEW and cardiovascular morbidity [7]. PEW has been considered to be not only due to poor nutritional intake but also the inflammatory process. Thus, malnutrition, inflammation, and atherosclerosis (MIA) syndrome is sometimes used for such situations in CKD-specific comorbidity [13]. Previous reports have suggested that inflammation often promotes a catabolic state, resulting in stimulating protein degradation, and suppressing protein synthesis, and causing a lower BMI in patients on HD [14,15]. In our study, we confirmed such relationships with easy methods. Moreover, combining both subjective and objective information on nutrition and inflammatory status might be important to estimate prognostic values accurately. 

Some methods have been used to estimate nutritional status. As screenings for daily clinical practice, simple and practical tools using routinely measured data may be ideal. The GNRI is calculated from both serum albumin, and the components of BMI have been developed as a simplified screening tool to assess nutritional risk [16]. This index has been reported to predict mortality or cardiovascular events in various settings. Notably, the GNRI is significantly associated with abdominal aortic calcification in non-dialyzed patients with chronic kidney disease. These results may suggest the utility of GNRI as a screening tool for predicting the severe vascular calcification in clinical practice [17]. In addition, we have already reported that GNRI accurately predicts CV events in HD patients [8]. GNRI has been also reported to predict CVD events including mortality due to CVD in patients with heart failure [18,19].

Recently, we have reported that predictability for mortality with GNRI is broadly comparable with criteria of PEW recommended by the International Society of Renal Nutrition and Metabolism (PEW-ISRNM), using serum albumin, BMI, mid-arm muscle circumference area, and daily protein-energy intake [20]. Therefore, GNRI may be a useful indicator instead of PEW-ISRNM to assess the malnutrition in HD patients. 

In our study, stratification by the positive number of risks factors could accurately predict mortality. Declined GNRI and elevated CRP levels were independently associated with abnormal ABI. In addition, CRP levels were also independently correlated with GNRI levels. All these factors are associated with each other; however, GNRI and ABI may more accurately reflect the nutritional status or vascular injury, respectively. The combination of these three factors can reflect not only inflammation but also malnutrition and atherosclerosis, and are therefore useful to assess PEW.

Some limitations should be discussed. First, this was a retrospective study with limited numbers of enrolled patients. Therefore, multi-center studies with large populations are needed to confirm our findings. Second, characteristics of our subjects such as body mass index, lipid profiles, and other factors were quite different from those in the Western countries. Third, ABI do not necessarily reflect presence of PAD. Patients on HD frequently have medial artery calcification, especially a high or normal ABI value. Fourth, we had no data on medications. Some medications such as statins, angiotensin-converting enzyme inhibitors, and angiotensin II receptor blockers might affect the results. Final, we only checked measurements at one time. Interval changes of the three parameters were not evaluated. In addition, revascularization for PAD before and after the enrollment was not considered. Additionally, values of blood pressure were not evaluated in the analysis. These limitations should be considered to interpret the results. 

## 5. Conclusions

In conclusion, ABI, GNRI, and CRP levels were closely associated with each other, and the combination of these variables strongly increased their predictive values for mortality due to CVD and all-cause mortality risk in HD patients. Thus, a relationship among malnutrition, inflammation, and atherosclerosis might manifest in this high-risk population.

## Figures and Tables

**Table 1:** Baseline patient characteristics in relation to presence of three risk factors: abnormal ankle brachial index (ABI), geriatric nutritional risk index (GNRI), and C-reactive protein (CRP).

Variables	All Patients (*n* = 973)	No Risk Factor (*n* = 259)	1 Risk Factors (*n* = 380)	2 Risk Factors (*n* = 257)	All Risk Factors (*n* = 77)	*p* Value
Male (%)	62.6	61.0	60.5	66.2	66.2	0.42
Age (years)	64 ± 12	62 ± 12	63 ± 11	66 ± 12	69 ± 11	<0.0001
Duration of HD (years)	2.0 (0.4–7.2)	1.8 (0.5–6.4)	2.2 (0.5–7.9)	2.0 (0.3–7.4)	2.1 (0.8–10.1)	0.41
Diabetes (%)	47.9	45.0	46.7	49.2	58.7	0.19
Hypertension (%)	74.9	77.4	72.8	76.4	72.2	0.54
Dyslipidemia (%)	14.4	13.9	12.9	17.3	13.9	0.54
Smoking (%)	25.4	21.4	26.9	27.8	23.9	0.38
Body mass index	21.6 ± 3.4	22.8 ± 3.6	22.0 ± 3.4	20.5 ± 2.9	19.2 ± 2.2	<0.0001
Previous CAD (%)	31.0	25.5	34.5	29.6	37.7	0.053
Previous stroke (%)	18.8	18.5	21.3	16.7	14.3	0.34
Hemoglobin (mg/dL)	10.5 ± 1.3	10.9 ± 1.0	10.6 ± 1.3	10.1 ± 1.2	9.6 ± 1.4	<0.0001
Albumin (g/dL)	3.6 ± 0.3	3.8 ± 0.2	3.6 ± 0.3	3.4 ± 0.4	3.3 ± 0.3	<0.0001
Creatinine (mg/dL)	9.6 ± 3.1	10.5 ± 3.6	9.9 ± 2.7	8.7 ± 2.8	7.8 ± 2.4	<0.0001
Calcium (mg/dL)	8.8 ± 0.8	8.7 ± 0.8	8.9 ± 0.9	8.6 ± 0.9	8.6 ± 0.9	0.37
Phosphate (mg/dL)	5.2 ± 1.3	5.4 ± 1.3	5.3 ± 1.3	5.1 ± 1.4	5.0 ± 1.3	0.012
Calcium x phosphate	46.1 ± 20.5	47.1 ± 12.0	47.9 ± 28.6	43.7 ± 13.3	43.2 ± 13.5	0.035
Total cholesterol (mg/dL)	161 ± 35	161 ± 34	163 ± 36	159 ± 35	159 ± 38	0.56
LDL cholesterol (mg/dL)	90 ± 30	87 ± 28	91 ± 30	89 ± 28	90 ± 30	0.59
HDL cholesterol (mg/dL)	44 ± 14	46 ± 14	43 ± 13	43 ± 14	43 ± 13	0.061
Triglyceride (mg/dL)	120 ± 85	118 ± 61	124 ± 77	117 ± 94	117 ± 86	0.78
CRP (mg/L)	1.6 (0.8–5.0)	0.8 (0.5–1.2)	1.6 (0.7–4.6)	3.8 (2.0–10.5)	5.0 (3.1–16.7)	<0.0001
Abnormal ABI (%)	34.1	0.0	27.9	58.0	100.0	<0.0001
GNRI	94.1 ± 8.8	99.7 ± 7.0	95.4 ± 0.4	89.3 ± 0.5	85.3 ± 0.9	<0.0001

**Table 2:** Associated factors for abnormal ABI (<0.9 or >1.4).

Variables	Univariate		Multivariate	
	OR (95% CI)	*p* value	OR (95% CI)	*p* value
Age	1.02 (1.01–1.03)	<0.0001	1.01 (1.00–1.03)	0.047
Diabetes	1.68 (1.2–2.209	0.0001	1.72 (1.30–2.26)	0.0001
Previous CAD	1.82 (1.38–2.41)	<0.0001	1.82 (1.36–2.43)	<0.0001
GNRI	0.97 (0.96–0.99)	0.0004	0.97 (0.96–0.99)	0.0009
CRP	1.16 (1.04–1.33)	0.011	1.13 (1.01–1.29)	0.043

**Table 3:** Relationship between GNRI and baseline variables by multivariate regression analysis.

Variables	Β	*p* Value
Age	−0.217	<0.0001
Hemoglobin	0.196	<0.0001
Creatinine	0.197	<0.0001
Phosphate	0.117	0.0020
CRP	−0.138	<0.0001
Abnormal ABI	−0.072	0.017

**Table 4:** Predictive value of abnormal ABI (<0.9 or >1.4), declined GNRI and elevated CRP for mortality due to cardiovascular disease (CVD) and all-cause mortality.

Variables	Univariate		Multivariate *	
	HR (95% CI)	*p* value	HR (95% CI)	*p* value
**Mortality Due to CVD**				
Abnormal ABI	1.96 (1.37–2.80)	0.0002	1.62 (1.13–2.32)	0.0096
GNRI < 91.2	1.83 (1.27–2.61)	0.0011	1.57 (1.06–2.33)	0.023
CRP > 1.9 mg/L	2.13 (1.48–3.08)	<0.0001	1.89 (1.31–2.77)	0.0007
**All-Cause Mortality**				
Abnormal ABI	2.07 (1.64–2.62)	<0.0001	1.68 (1.33–2.14)	<0.0001
GNRI < 91.2	2.47 (1.96–3.13)	<0.0001	2.12 (1.64–2.74)	<0.0001
CRP > 1.9 mg/L	2.34 (1.85–2.99)	<0.0001	2.02 (1.58–2.60)	<0.0001

* adjusted for male, age, diabetes, previous CAD, previous stroke, hemoglobin, creatinine, and phosphate as baseline variables with *p* < 0.05 by univariate analysis.

**Table 5:** Combined predictive value of abnormal ABI, GNRI, and CRP for CVD- and all-cause mortality.

Variables	Univariate		Multivariate *	
	HR (95% CI)	*p* value	HR (95% CI)	*p* value
**Mortality Due to CVD** (vs. no risk factor)				
Any single risk factor	2.43 (1.35–4.67)	0.0023	2.28 (1.26–4.42)	0.0054
Any two risk factors	4.05 (2.25–7.82)	<0.0001	3.61 (1.97–7.05)	<0.0001
All risk factors	7.38 (3.63–15.4)	<0.0001	5.26 (2.51–11.3)	<0.0001
**All-Cause Mortality** (vs. no risk factor)				
Any single risk factor	2.46 (1.62–3.88)	<0.0001	2.55 (1.66–4.06)	<0.0001
Any two risk factors	5.04 (3.34–7.88)	<0.0001	4.59 (3.00–7.29)	<0.0001
All risk factors	10.6 (6.64–17.2)	<0.0001	7.97 (4.88–13.2)	<0.0001

* adjusted for male, age, diabetes, previous CAD, previous stroke, hemoglobin, creatinine, and phosphate as baseline variables with *p* < 0.05 by univariate analysis.

**Table 6:** Discrimination of each predicting models for mortality using C-index, net reclassification improvement (NRI), and integrated discrimination improvement (IDI).

Variables	C-Index	*p* Value	NRI	*p* Value	IDI	*p* Value
**Mortality Due to CVD**						
Established risk factors	0.654	Reference	Reference		Reference	
+abnormal ABI	0.710	0.012	0.534	<0.0001	0.018	<0.0001
+GNRI	0.711	0.0027	0.415	<0.0001	0.017	<0.0001
+CRP	0.692	0.0083	0.388	<0.0001	0.015	0.0049
+All factors	0.765	<0.0001	0.518	<0.0001	0.036	<0.0001
+all factors vs. +abnormal ABI	0.055 *	0.0027	0.470	<0.0001	0.028	<0.0001
+all factors vs. +GNRI	0.054 *	0.0051	0.348	0.0002	0.019	<0.0001
+all factors vs. +CRP	0.073 *	0.0004	0.459	<0.0001	0.031	<0.0001
**All-Cause Mortality**						
Established risk factors	0.669	Reference	Reference		Reference	
+abnormal ABI	0.718	0.0008	0.511	<0.0001	0.040	<0.0001
+GNRI	0.737	<0.0001	0.412	<0.0001	0.065	<0.0001
+CRP	0.710	<0.0001	0.462	<0.0001	0.036	<0.0001
+All factors	0.794	<0.0001	0.587	<0.0001	0.132	<0.0001
+all factors vs. +abnormal ABI	0.076 *	<0.0001	0.685	<0.0001	0.092	<0.0001
+all factors vs. +GNRI	0.057 *	0.0002	0.343	<0.0001	0.066	<0.0001
+all factors vs. +CRP	0.084 *	<0.0001	0.553	<0.0001	0.095	<0.0001

Established risk factors included male, age, diabetes, previous CAD, previous stroke, hemoglobin, creatinine, and phosphate as baseline variables, with *p* < 0.05 by univariate analysis. * Estimated differences between two groups.
